# Supplementary material for: Effect of Ethylene Oxide Exposure on Sleep Health: Using NHANES Data from 2015 to 2020
Source: Healthcare (Basel). 2024 Dec 11;12(24):2499. doi: 10.3390/healthcare12242499 (PMC11728014; doi:10.3390/healthcare12242499)
Supplement: Supplementary file 1 [file healthcare-12-02499-s001.zip › healthcare-3328561-supplementary.pdf]

Table S1. Sex-specific adjusted odds ratios of the sleep health according to ethylene oxide concentration

Variables	*Adjusted OR (95% CI) for sleep health							
	Continuous, ln		Categorical, quartiles					
			Q1	Q2		Q3		Q4	
Men									
Sleep duration, hours/day (last 12 months)									
<6, very insufficient	1.173	(1.009, 1.365)	1.000	2.675	(1.315, 5.439)	2.525	(1.251, 5.096)	2.589	(1.285, 5.216)
<7, insufficient	1.067	(0.931, 1.222)	1.000	0.898	(0.557, 1.449)	0.928	(0.574, 1.499)	1.328	(0.783, 2.252)
≥9, excessive	0.950	(0.810, 1.115)	1.000	1.132	(0.611, 2.096)	0.887	(0.461, 1.708)	0.661	(0.341, 1.284)
Sleep quality (last 12 months)									
Snoring, ≥1/week	1.114	(0.968, 1.283)	1.000	0.862	(0.526, 1.413)	1.364	(0.870, 2.141)	1.576	(0.909, 2.733)
Snorting, gasping or stopping breathing, ≥1/week	1.065	(0.931, 1.220)	1.000	0.535	(0.347, 0.825)	0.862	(0.530, 1.402)	1.074	(0.617, 1.869)
Day sleepiness	1.033	(0.916, 1.165)	1.000	0.858	(0.531, 1.385)	1.266	(0.776, 2.068)	1.240	(0.766, 2.007)
Sleep problems	1.046	(0.932, 1.175)	1.000	1.366	(0.887, 2.102)	0.907	(0.608, 1.352)	1.473	(0.929, 2.335)
Obstructive sleep apnea	0.936	(0.835, 1.050)	1.000	0.578	(0.373, 0.896)	0.938	(0.608, 1.449)	0.661	(0.420, 1.041)
Women									
Sleep duration, hours/day (last 12 months)									
<6, very insufficient	1.386	(1.073, 1.788)	1.000	1.471	(0.616, 3.515)	1.494	(0.793, 2.815)	1.582	(0.947, 2.643)
<7, insufficient	1.260	(1.075, 1.477)	1.000	1.347	(0.732, 2.478)	1.468	(0.864, 2.493)	1.560	(0.878, 2.771)
≥9, excessive	1.133	(0.965, 1.330)	1.000	1.232	(0.782, 1.941)	1.141	(0.763, 1.704)	1.802	(1.057, 3.073)
Sleep quality (last 12 months)									
Snoring, ≥1/week	0.979	(0.839, 1.144)	1.000	0.909	(0.564, 1.466)	0.882	(0.519, 1.501)	0.893	(0.576, 1.385)
Snorting, gasping or stopping breathing, ≥1/week	1.152	(1.010, 1.313)	1.000	1.336	(0.714, 2.498)	1.498	(0.852, 2.493)	1.265	(0.734, 2.182)
Day sleepiness	1.057	(0.897, 1.246)	1.000	1.205	(0.714, 2.033)	1.697	(1.059, 2.719)	1.152	(0.697, 1.903)
Sleep problems	1.065	(0.892, 1.272)	1.000	0.840	(0.612, 1.154)	1.374	(0.857, 2.204)	1.497	(0.903, 2.481)
Obstructive sleep apnea	0.991	(0.809, 1.212)	1.000	1.096	(0.764, 1.573)	1.038	(0.662, 1.626)	1.032	(0.618, 1.725)

*Adjusted for age, sex, race. socioeconomic status (economic status, marital status, job status), comorbidities (history of cardiovascular disease and cancer, hypertension, diabetes mellitus, depressive symptom), life style factors (body mass index, physical activity, sedentary activity, caffeine consumption, alcohol drinking, cotinine)

Table S2. Age-specific adjusted odds ratios of the sleep health according to ethylene oxide concentration

Variables	*Adjusted OR (95% CI) for sleep health								
	Continuous, ln		Categorical, quartiles						
			Q1	Q2		Q3		Q4	
**<40**									
Sleep duration, hours/day (last 12 months)									
<6, very insufficient	1.392	(1.113, 1.740)	1.000	0.930	(0.590, 1.466)	0.952	(0.573, 1.582)	1.458	(1.319, 1.800)
<7, insufficient	0.781	(0.638, 0.957)	1.000	1.243	(0.860, 1.797)	1.140	(0.745, 1.744)	1.402	(0.644, 2.961)
≥9, excessive	0.956	(0.761, 1.201)	1.000	0.820	(0.522, 1.286)	0.955	(0.580, 1.571)	0.960	(0.407, 2.262)
Sleep quality (last 12 months)									
Snoring, ≥1/week	0.930	(0.757, 1.142)	1.000	1.151	(0.787, 1.684)	1.534	(0.981, 2.396)	1.378	(0.641, 2.960)
Snorting, gasping or stopping breathing, ≥1/week	1.068	(0.830, 1.375)	1.000	0.938	(0.599, 1.467)	0.826	(0.487, 1.404)	0.673	(0.257, 1.761)
Day sleepiness	1.086	(0.805, 1.207)	1.000	0.978	(0.660, 1.450)	0.981	(0.625, 1.539)	1.431	(0.671, 3.056)
Sleep problems	1.066	(0.793, 1.170)	1.000	0.993	(0.689, 1.432)	0.857	(0.560, 1.311)	1.630	(0.798, 3.469)
Obstructive sleep apnea	1.015	(0.830, 1.242)	1.000	0.912	(0.631, 1.319)	1.065	(0.696, 1.632)	1.772	(0.362, 1.651)
**40-49**									
Sleep duration, hours/day (last 12 months)									
<6, very insufficient	0.983	(0.729, 1.326)	1.000	0.799	(0.433, 1.440)	1.169	(0.613, 2.232)	1.361	(0.414, 4.473)
<7, insufficient	1.121	(0.852, 1.476)	1.000	0.943	(0.562, 1.581)	0.844	(0.467, 1.525)	0.556	(0.184, 1.678)
≥9, excessive	0.829	(0.578, 1.189)	1.000	1.589	(0.782, 3.231)	1.089	(0.466, 2.546)	1.858	(0.444, 7.771)
Sleep quality (last 12 months)									
Snoring, ≥1/week	0.818	(0.595, 1.124)	1.000	0.924	(0.512, 1.667)	0.917	(0.469, 1.796)	1.999	(0.545, 7.334)
Snorting, gasping or stopping breathing, ≥1/week	1.115	(0.820, 1.516)	1.000	0.733	(0.390, 1.378)	1.332	(0.682, 2.602)	1.107	(0.311, 3.942)
Day sleepiness	1.203	(0.870, 1.664)	1.000	0.801	(0.429, 1.492)	0.953	(0.477, 1.907)	1.516	(1.138, 1.943)
Sleep problems	1.069	(0.658, 1.149)	1.000	1.061	(0.634, 1.787)	1.308	(0.722, 2.370)	1.664	(0.545, 5.079)
Obstructive sleep apnea	1.024	(0.752, 1.306)	1.000	0.861	(0.516, 1.435)	0.796	(0.442, 1.433)	1.396	(0.453, 4.135)
**50-59**									
Sleep duration, hours/day (last 12 months)									
<6, very insufficient	1.143	(0.858, 1.523)	1.000	1.038	(0.599, 1.796)	0.906	(0.487, 1.686)	0.824	(0.252, 2.690)
<7, insufficient	0.961	(0.740, 1.249)	1.000	0.665	(0.402, 1.100)	0.862	(0.488, 1.522)	0.698	(0.242, 2.017)
≥9, excessive	0.887	(0.641, 1.226)	1.000	1.119	(0.987, 4.449)	0.936	(0.717, 3.731)	2.652	(0.686, 9.248)
Sleep quality (last 12 months)									

Snoring, ≥1/week	0.995	(0.742, 1.335)	1.000	0.688	(0.377, 1.256)	0.923	(0.470, 1.812)	0.731	(0.221, 2.421)
Snorting, gasping or stopping breathing, ≥1/week	1.245	(0.926, 1.673)	1.000	0.737	(0.418, 1.299)	0.858	(0.457, 1.610)	0.433	(0.127, 1.473)
Day sleepiness	1.094	(0.805, 1.485)	1.000	1.408	(0.774, 2.563)	0.844	(0.424, 1.679)	0.775	(0.218, 2.763)
Sleep problems	1.192	(0.909, 1.563)	1.000	0.896	(0.540, 1.487)	0.702	(0.395, 1.248)	0.810	(0.274, 2.393)
Obstructive sleep apnea	1.026	(0.791, 1.331)	1.000	1.053	(0.638, 1.740)	1.118	(0.636, 1.968)	1.287	(0.725, 2.490)
**60-69**									
Sleep duration, hours/day (last 12 months)									
<6, very insufficient	1.046	(0.761, 1.438)	1.000	0.739	(0.431, 1.268)	0.799	(0.427, 1.494)	0.606	(0.178, 2.062)
<7, insufficient	0.790	(0.599, 1.041)	1.000	1.906	(1.179, 3.082)	1.291	(0.742, 2.249)	2.585	(0.898, 7.441)
≥9, excessive	1.291	(0.951, 1.755)	1.000	0.535	(0.294, 0.975)	0.926	(0.490, 1.751)	0.498	(0.152, 1.630)
Sleep quality (last 12 months)									
Snoring, ≥1/week	1.025	(0.763, 1.377)	1.000	0.808	(0.461, 1.417)	0.717	(0.382, 1.347)	0.792	(0.251, 2.496)
Snorting, gasping or stopping breathing, ≥1/week	1.373	(1.079, 1.926)	1.000	0.899	(0.537, 1.505)	1.431	(1.224, 1.829)	1.234	(1.061, 1.888)
Day sleepiness	1.009	(0.649, 1.272)	1.000	1.446	(0.821, 2.547)	1.500	(0.768, 2.928)	1.347	(0.366, 4.960)
Sleep problems	1.081	(0.668, 1.161)	1.000	1.168	(0.732, 1.862)	0.899	(0.520, 1.555)	1.853	(0.624, 5.353)
Obstructive sleep apnea	1.298	(1.087, 1.706)	1.000	0.720	(0.447, 1.159)	1.166	(1.028, 1.810)	1.306	(1.107, 1.875)
**≥ 70**									
Sleep duration, hours/day (last 12 months)									
<6, very insufficient	1.030	(0.693, 1.531)	1.000	1.354	(0.735, 2.492)	1.113	(0.494, 2.507)	2.153	(1.482, 9.616)
<7, insufficient	0.956	(0.681, 1.342)	1.000	0.659	(0.412, 1.052)	0.618	(0.327, 1.167)	0.816	(0.228, 2.921)
≥9, excessive	1.050	(0.717, 1.537)	1.000	1.324	(0.793, 2.210)	1.620	(0.811, 3.236)	1.658	(0.149, 2.901)
Sleep quality (last 12 months)									
Snoring, ≥1/week	0.850	(0.598, 1.209)	1.000	1.309	(0.806, 2.125)	1.909	(0.981, 3.714)	2.316	(0.609, 8.804)
Snorting, gasping or stopping breathing, ≥1/week	1.250	(0.821, 1.902)	1.000	0.663	(0.377, 1.166)	0.670	(0.308, 1.456)	0.632	(0.123, 3.250)
Day sleepiness	1.032	(0.677, 1.428)	1.000	1.014	(0.604, 1.703)	0.877	(0.431, 1.781)	1.320	(0.322, 5.403)
Sleep problems	1.012	(0.705, 1.394)	1.000	0.944	(0.593, 1.503)	0.978	(0.520, 1.840)	1.726	(0.477, 6.249)
Obstructive sleep apnea	1.097	(0.775, 1.552)	1.000	1.138	(0.709, 1.826)	1.082	(0.568, 2.058)	1.165	(0.781, 3.284)

*Adjusted for age, sex, race. socioeconomic status (economic status, marital status, job status), comorbidities (history of cardiovascular disease and cancer, hypertension, diabetes mellitus, depressive symptom), life style factors (body mass index, physical activity, sedentary activity, caffeine consumption, alcohol drinking, cotinine)

Table S3. Marital status-specific adjusted odds ratios of the sleep health according to ethylene oxide concentration

Variables	*Adjusted OR (95% CI) for sleep health								
	Continuous, ln		Categorical, quartiles						
			Q1	Q2		Q3		Q4	
**Married/Cohabiting**									
Sleep duration, hours/day (last 12 months)									
<6, very insufficient	1.304	(1.089, 1.560)	1.000	0.966	(0.715, 1.306)	0.852	(0.594, 1.222)	0.552	(0.269, 1.136)
<7, insufficient	1.051	(1.029, 2.015)	1.000	1.109	(0.860, 1.430)	1.097	(0.807, 1.491)	1.383	(0.758, 2.524)
≥9, excessive	0.934	(0.773, 1.128)	1.000	0.890	(0.644, 1.231)	1.086	(0.742, 1.590)	1.232	(0.598, 2.544)
Sleep quality (last 12 months)									
Snoring, ≥1/week	0.940	(0.789, 1.120)	1.000	1.048	(0.782, 1.405)	1.208	(0.849, 1.719)	1.304	(0.655, 2.554)
Snorting, gasping or stopping breathing, ≥1/week	1.346	(1.121, 1.616)	1.000	1.164	(1.149, 1.885)	1.230	(1.061, 1.926)	1.301	(1.144, 1.628)
Day sleepiness	0.996	(0.838, 1.184)	1.000	1.170	(0.873, 1.567)	1.184	(0.833, 1.682)	1.344	(0.687, 2.631)
Sleep problems	1.016	(0.872, 1.185)	1.000	0.998	(0.775, 1.284)	0.905	(0.666, 1.228)	1.322	(0.718, 2.354)
Obstructive sleep apnea	1.121	(0.960, 1.308)	1.000	0.890	(0.692, 1.146)	0.784	(0.578, 1.063)	0.637	(0.350, 1.157)
**Divorced/Widowed/Separated**									
Sleep duration, hours/day (last 12 months)									
<6, very insufficient	1.083	(0.775, 1.246)	1.000	1.109	(0.662, 1.859)	1.083	(0.601, 1.952)	1.845	(0.713, 4.775)
<7, insufficient	0.936	(0.749, 1.171)	1.000	0.650	(0.415, 1.016)	0.677	(0.403, 1.137)	0.648	(0.266, 1.581)
≥9, excessive	1.120	(0.869, 1.444)	1.000	1.623	(0.951, 2.771)	1.592	(0.867, 2.925)	0.949	(0.333, 2.705)
Sleep quality (last 12 months)									
Snoring, ≥1/week	0.837	(0.668, 1.049)	1.000	0.938	(0.597, 1.474)	1.068	(0.632, 1.807)	1.524	(0.615, 3.778)
Snorting, gasping or stopping breathing, ≥1/week	1.049	(0.798, 1.378)	1.000	1.523	(0.874, 2.655)	1.018	(0.528, 1.965)	1.038	(0.893, 2.742)
Day sleepiness	1.075	(0.870, 1.429)	1.000	1.041	(0.623, 1.741)	1.079	(0.543, 1.767)	1.107	(0.259, 1.935)
Sleep problems	0.963	(0.768, 1.207)	1.000	1.010	(0.645, 1.580)	0.964	(0.572, 1.623)	1.445	(0.586, 3.563)
Obstructive sleep apnea	0.948	(0.759, 1.182)	1.000	1.071	(0.684, 1.677)	1.102	(0.656, 1.849)	1.260	(0.520, 3.055)
**Never married**									
Sleep duration, hours/day (last 12 months)									
<6, very insufficient	1.063	(0.718, 1.291)	1.000	1.040	(0.321, 1.103)	1.094	(0.517, 1.901)	1.511	(0.497, 4.588)
<7, insufficient	0.965	(0.741, 1.255)	1.000	1.306	(0.792, 2.154)	0.822	(0.465, 1.453)	0.784	(0.291, 2.118)
≥9, excessive	1.074	(0.800, 1.443)	1.000	1.169	(0.639, 2.139)	1.358	(0.694, 2.657)	0.955	(0.312, 2.921)
Sleep quality (last 12 months)									
Snoring, ≥1/week	1.020	(0.783, 1.328)	1.000	1.078	(0.654, 1.779)	1.456	(0.815, 2.601)	1.124	(0.415, 3.041)
Snorting, gasping or stopping breathing, ≥1/week	0.943	(0.687, 1.293)	1.000	1.013	(0.516, 1.989)	1.081	(0.514, 2.274)	1.983	(0.587, 6.703)
Day sleepiness	0.914	(0.688, 1.213)	1.000	0.818	(0.478, 1.400)	0.675	(0.360, 1.263)	1.356	(0.468, 3.930)
Sleep problems	0.830	(0.636, 1.082)	1.000	0.953	(0.578, 1.570)	0.862	(0.485, 1.533)	3.013	(1.101, 8.247)
Obstructive sleep apnea	1.121	(0.854, 1.471)	1.000	0.987	(0.592, 1.686)	1.109	(0.614, 2.005)	0.692	(0.245, 1.952)

*Adjusted for age, sex, race. socioeconomic status (economic status, marital status, job status), comorbidities (history of cardiovascular disease and cancer, hypertension, diabetes mellitus, depressive symptom), life style factors (body mass index, physical activity, sedentary activity, caffeine consumption, alcohol drinking, cotinine)

Table S4. Job status-specific adjusted odds ratios of the sleep health according to ethylene oxide concentration

Variables	*Adjusted OR (95% CI) for sleep health								
	Continuous, ln		Categorical, quartiles						
			Q1	Q2		Q3		Q4	
**With a job or business**									
Sleep duration, hours/day (last 12 months)									
<6, very insufficient	1.051	(0.890, 1.242)	1.000	0.901	(0.669, 1.215)	1.110	(0.787, 1.565)	1.207	(0.635, 2.296)
<7, insufficient	0.953	(0.819, 1.110)	1.000	1.044	(0.801, 1.360)	0.939	(0.688, 1.280)	0.874	(0.488, 1.563)
≥9, excessive	1.010	(0.816, 1.224)	1.000	1.112	(0.766, 1.615)	0.984	(0.638, 1.571)	0.994	(0.461, 2.142)
Sleep quality (last 12 months)									
Snoring, ≥1/week	0.929	(0.789, 1.067)	1.000	1.001	(0.749, 1.337)	1.083	(0.771, 1.520)	1.076	(0.578, 2.001)
Snorting, gasping or stopping breathing, ≥1/week	1.129	(0.934, 1.364)	1.000	0.872	(0.639, 1.191)	0.870	(0.599, 1.262)	0.579	(0.274, 1.222)
Day sleepiness	0.914	(0.770, 1.085)	1.000	1.109	(0.819, 1.502)	1.102	(0.771, 1.573)	1.645	(0.862, 3.140)
Sleep problems	0.922	(0.792, 1.073)	1.000	1.118	(0.860, 1.454)	1.012	(0.733, 1.366)	1.742	(1.075, 3.113)
Obstructive sleep apnea	1.058	(0.908, 1.233)	1.000	0.961	(0.741, 1.247)	0.850	(0.624, 1.157)	0.694	(0.387, 2.093)
**Looking for work/Not working**									
Sleep duration, hours/day (last 12 months)									
<6, very insufficient	1.270	(1.043, 1.546)	1.000	1.028	(0.694, 1.523)	0.797	(0.504, 1.261)	1.095	(1.071, 1.556)
<7, insufficient	1.120	(1.060, 1.974)	1.000	0.998	(0.734, 1.357)	0.929	(0.644, 1.340)	1.326	(0.677, 2.596)
≥9, excessive	1.015	(0.850, 1.211)	1.000	1.019	(0.733, 1.417)	1.342	(0.914, 1.971)	1.036	(0.512, 2.093)
Sleep quality (last 12 months)									
Snoring, ≥1/week	0.899	(0.745, 1.061)	1.000	1.089	(0.790, 1.501)	1.458	(0.998, 2.145)	1.643	(0.814, 3.317)
Snorting, gasping or stopping breathing, ≥1/week	1.209	(1.097, 1.466)	1.000	0.824	(0.577, 1.170)	0.732	(0.480, 1.115)	1.134	(1.053, 1.208)
Day sleepiness	1.117	(0.928, 1.345)	1.000	1.098	(0.781, 1.545)	0.962	(0.640, 1.445)	1.068	(0.363, 1.627)
Sleep problems	1.004	(0.845, 1.195)	1.000	0.897	(0.659, 1.219)	0.839	(0.582, 1.210)	1.457	(0.870, 2.872)
Obstructive sleep apnea	1.077	(0.910, 1.274)	1.000	0.951	(0.702, 1.293)	1.030	(0.691, 1.436)	1.039	(0.431, 1.638)

*Adjusted for age, sex, race. socioeconomic status (economic status, marital status, job status), comorbidities (history of cardiovascular disease and cancer, hypertension, diabetes mellitus, depressive symptom), life style factors (body mass index, physical activity, sedentary activity, caffeine consumption, alcohol drinking, cotinine)
